# Implementation of a workplace intervention using financial rewards to promote adherence to physical activity guidelines: a feasibility study

**DOI:** 10.1186/s12889-017-4931-2

**Published:** 2017-12-01

**Authors:** Elena Losina, Savannah R. Smith, Ilana M. Usiskin, Kristina M. Klara, Griffin L. Michl, Bhushan R. Deshpande, Heidi Y. Yang, Karen C. Smith, Jamie E. Collins, Jeffrey N. Katz

**Affiliations:** 1 Orthopaedic and Arthritis Center for Outcomes Research, Policy and Innovation Evaluation in Orthopaedic Treatments (PIVOT) Center, Department of Orthopaedic Surgery, Brigham and Women’s Hospital, 75 Francis Street, BTM 5-016, Boston, MA 02115 USA; 2 Harvard Medical School, Boston, MA USA; 3 Division of Rheumatology, Immunology, and Allergy, Brigham and Women’s Hospital, Boston, MA USA; 4 Department of Biostatistics, Boston University School of Public Health, Boston, MA USA; 5 Departments of Epidemiology and Environmental Health, Harvard T. H. Chan School of Public Health, Boston, MA USA

**Keywords:** Physical activity, Workplace, Exercise, Financial incentives

## Abstract

**Background:**

We designed and implemented the Brigham and Women’s Wellness Initiative (B-Well), a single-arm study to examine the feasibility of a workplace program that used individual and team-based financial incentives to increase physical activity among sedentary hospital employees.

**Methods:**

We enrolled sedentary, non-clinician employees of a tertiary medical center who self-reported low physical activity. Eligible participants formed or joined teams of three members and wore Fitbit Flex activity monitors for two pre-intervention weeks followed by 24 weeks during which they could earn monetary rewards. Participants were rewarded for increasing their moderate-to-vigorous physical activity (MVPA) by 10% from the previous week or for meeting the Centers for Disease Control and Prevention (CDC) physical activity guidelines (150 min of MVPA per week). Our primary outcome was the proportion of participants meeting weekly MVPA goals and CDC physical activity guidelines. Secondary outcomes included Fitbit-wear adherence and factors associated with meeting CDC guidelines more consistently.

**Results:**

B-Well included 292 hospital employees. Participants had a mean age of 38 years (SD 11), 83% were female, 38% were obese, and 62% were non-Hispanic White. Sixty-three percent of participants wore the Fitbit ≥4 days per week for ≥20 weeks. Two-thirds were satisfied with the B-Well program, with 79% indicating that they would participate again. Eighty-six percent met either their personal weekly goal or CDC physical activity guidelines for at least 6 out of 24 weeks, and 52% met their goals or CDC physical activity guidelines for at least 12 weeks. African Americans, non-obese subjects, and those with lower impulsivity scores reached CDC guidelines more consistently.

**Conclusions:**

Our data suggest that a financial incentives-based workplace wellness program can increase MVPA among sedentary employees. These results should be reproduced in a randomized controlled trial.

**Trial registration:**

Clinicaltrials.gov, NCT02850094. Registered July 27, 2016 [retrospectively registered].

## Background

Physical activity (PA) is associated with enhanced quality of life in healthy individuals and improvement in health in those with cardiovascular disease, musculoskeletal disorders, some cancers, depression, and diabetes [1–5]. Higher PA levels are associated with reduced healthcare utilization, lower medical costs, and fewer work absences [6, 7]. Public health agencies recommend at least 150 min of moderate or 75 min of vigorous PA per week; however, only about half of American adults meet these guidelines [8, 9]. Identifying effective strategies for increasing PA and adherence to PA recommendations could therefore have substantial health implications.

Behavioral economic theory provides a framework for understanding unhealthy behaviors by acknowledging that people do not behave rationally in all circumstances [10, 11]. Individuals commonly place more value on present comfort than on future health benefits [12, 13]. Financial incentive interventions combat this fallacy by providing an immediate benefit to engaging in healthy behaviors [11]. Financial incentives have been shown to be effective in encouraging healthy behavioral changes [14–16], including increasing PA [17–20]. Financial incentives increased walking in an older sedentary population [18], and a meta-analysis found that they had a modest effect on short-term exercise, increasing exercise session attendance by 11.55% [19].

Workplace interventions to promote PA have increased in popularity. This is particularly true in the United States, where employers are a major source of health insurance and thus have reason to promote healthy behaviors in their employees [21]. A randomized controlled trial in a workplace setting found that financial incentives effectively promoted walking; the group receiving incentives that were framed as financial losses averaged 5880 steps/day compared to 5031 in the control group [22]. Structuring workplace financial incentives around both individual and team rewards also improves intervention outcomes [23].

Given the potential public health benefits of improved adherence to PA guidelines, the increasing use of personal accelerometers, and the growing number of workplace interventions offered across the US, we designed the Brigham and Women’s Wellness Initiative (B-Well). B-Well was a 26-week program designed to investigate the feasibility of individual and team-based financial incentives to increase objectively-measured PA among sedentary hospital employees. Here, we report the implementation and outcomes of B-Well with the goals of both offering insight into how a financial incentives program can be implemented in the workplace and framing appropriate expectations for program outcomes.

## Methods

### Setting, eligibility, and enrollment

B-Well was conducted at Brigham and Women’s Hospital, an academic medical center in Boston, Massachusetts. The study ran from July 2014 to June 2015 and was funded to include 300 participants. All participants received the financial incentives intervention; there was no control arm. Employees were recruited via a limited number of hospital-wide emails and advertisements. Eligible individuals were older than 21 years, reported ≤30 min of exercise per week, and had access to a computer. Weekly minutes of exercise were assessed with a question asking participants if they exercised for 30 min on 0, 1, 2, 3, or ≥4 days of the week. Physicians and nurses were not eligible for participation, as they had access to other similar PA programs. Pregnant women were excluded.

Eligible employees formed three-person teams, created independently or by B-Well staff, to provide peer support. Each team met with study staff to review the study protocol, PA monitoring schedule, and financial incentive details. At this meeting, each participant was given a Fitbit Flex activity monitor (Fitbit Inc., San Francisco, California) and instructions for its use.

### Baseline assessments

Trained research staff collected health data at the baseline visit. Participants provided written informed consent and completed an online questionnaire containing questions related to general health, healthcare utilization, quality of life (measured by EQ-5D), and perceptions of PA. Participants self-reported comorbidities including heart disease, hypertension, diabetes, osteoarthritis, and others. Musculoskeletal disease was assessed with a validated musculoskeletal index [24].

Participants also completed an assessment to determine their degree of delay discounting. Delay discounting, a measure of impulsivity, describes the extent to which individuals prefer immediate, smaller rewards to larger, long-term gains [12, 25, 26]. Based on choices between receiving a specified monetary amount now or receiving a larger amount later, participants were assigned a value on a delay discounting index, with higher values indicating greater preference for immediate rewards. We used a method developed by Kirby and Petry to estimate the index [27].

### Intervention and financial incentives structure

Participants were asked to wear their Fitbits continuously for 26 weeks. During the first 2 weeks, participants were in the pre-intervention period and were compensated for wearing the Fitbit. They received $20 for wearing the Fitbit for at least 10 days and an additional $10 for wearing it for all 14 days. Teams in which all members met the 10 day criterion received an additional $10 per member. Results from these 2 weeks were considered baseline levels of PA.

Financial incentives, chosen to be meaningful but not coercive, were available for the subsequent 24 weeks. Participants could receive $10 per week for increasing minutes of moderate-to-vigorous PA (MVPA) by at least 10% from the preceding week or for meeting Centers for Disease Control and Prevention (CDC) PA guidelines (150 min of moderate PA per week) [8]. Individuals meeting their MVPA goal every week for one, three, or 6 months received an additional $15, $25, or $50 respectively. If all team members successfully met their MVPA goals, each individual’s reward was doubled that week. In total, participants could receive up to $860 by meeting MVPA goals. These amounts fall within the range identified by a 2013 systematic review on financial incentives for exercise session attendance, which noted that successful programs had weekly incentives ranging from $2.79 to $46.82 [19].

Participants received individualized weekly emails during the intervention detailing their levels of recent MVPA, their weekly goals, and their recent rewards. While participants could view activity data (e.g. step counts) on the Fitbit website, this data did not include the minutes of MVPA that counted toward their goals. Participants could visit the B-Well study website to monitor current and past individual and team progress.

### Measuring MVPA

The Fitbit Flex is a commercial accelerometer that records the number of steps taken each minute. We chose the Fitbit Flex because it presents an adequate trade-off between validity and expense; it is less expensive than medical grade accelerometers and has moderate validity for measuring step count and physical activity [28–30]. We downloaded minute-by-minute step counts weekly and defined thresholds of 100 and 175 steps/min for moderate and vigorous PA, respectively. The threshold of 100 steps/min was selected as it corresponds with an intensity of ≥3 metabolic equivalents of task (METs) [31, 32]. As there is limited data on the association between step count and vigorous activity, we used the conservative threshold of 175 steps/min. We weighted minutes of vigorous activity as twice that of moderate activity, as CDC guidelines recommend either 150 min of moderate activity, 75 min of vigorous activity, or an equivalent combination per week [8]. Days with fewer than 10 h of wear time were excluded from analysis. To count towards the weekly goal, activity had to be performed in bouts of at least 10 min, in accordance with the CDC PA guidelines [8]. Within a single bout, we allowed up to two ‘grace’ minutes during which the participant’s step count could fall below the thresholds.

Our algorithm identified the start of potential bouts of moderate PA either as a minute with more than 100 steps preceded by a minute of fewer than 100 steps or as 100 steps in the first minute of the day. Bout length was defined as the number of consecutive minutes during which the step count met the 100 steps/min threshold before the two grace minutes were exceeded. Bouts of fewer than 10 min were not counted. The algorithm then conducted consecutive pair-wise comparisons of each bout of at least 10 min. When two bouts overlapped, the shorter of the two was discarded and the pair-wise comparison continued until no bouts were overlapping. The algorithm repeated with a 175 steps/min threshold to identify bouts of vigorous PA.

### Outcomes

We assessed intervention efficacy on several domains. First, we calculated the average number of minutes spent in bouts of MVPA on a weekly basis and compared these values during the intervention period to those during the pre-intervention period. Second, we measured Fitbit adherence among enrolled participants by counting weeks in which participants wore the Fitbit for at least 10 h per day for at least 4 days of the week. We defined adherent Fitbit wearers as participants with at least 20 adherent intervention weeks (out of 24). Third, we evaluated the number and proportion of study participants who met their weekly MVPA goals or CDC PA guidelines during the intervention.

### Statistical analysis

We employed generalized linear modeling to determine factors associated with the number of weeks meeting CDC PA guidelines. The multivariable analyses included demographic and clinical variables and the delay discounting index, which was divided into the highest quartile (the highest degree of discounting future benefits) vs. the lowest three quartiles combined. Based on the multivariable model results, we estimated the adjusted mean number of intervention weeks during which subjects achieved MVPA levels consistent with CDC PA guidelines. We initially built bivariate models; covariates that reached a significance level of α = 0.15 were advanced to multivariable models. We used intraclass correlation to measure within-team correlation for meeting CDC guidelines. We calculated within-team correlation for minutes of MVPA over time to examine whether within team correlation was based on selection (active participants form teams with active participants) or adaption (teams are mixed with active and inactive participants at the start and correlation increases over time). All statistical analyses were done in SAS 9.4.

## Results

### Sample characteristics

Seven hundred and seventy-seven employees were screened, 398 were eligible, and 300 were enrolled in the study (the remaining 98 were placed on a waiting list). Eight subjects did not wear their Fitbits at any point, and their data were not analyzed. Nine participants formally withdrew over the course of the program; six did so because they left their positions with the hospital. The final analytic sample consisted of 292 hospital employees who were 83% female and had a mean age of 38 years (standard deviation [SD] 11). The majority of participants (78%) created a team independently. Sixty-two percent of participants were white, 38% were obese, and 75% had at least a Bachelor’s degree. Thirty-four percent reported being limited to walking less than 20 blocks (one mile) (Table 1).

**Table 1:** Sample Characteristics

Characteristic	n (%)
Sample Size	292
Age - Mean (SD)	38 (11)
Female	250 (83%)
Race/Ethnicity	
White	181 (62%)
Black	41 (14%)
Hispanic	19 (7%)
Asian	30 (10%)
Other	21 (7%)
Education	
High school or less	14 (5%)
Some college	57 (20%)
Graduated from college	218 (75%)
Annual Household Income	
$0 – $59,000	89 (30%)
$60,000 – $99,000	84 (29%)
More than $100,000	116 (40%)
Living Situation	
Alone	34 (12%)
With spouse/partner	174 (60%)
With other family or friends	83 (29%)
Weekly Alcohol Consumption	
None	95 (33%)
1–2 Drinks per Week	113 (39%)
3+ Drinks per Week	80 (28%)
Body Mass Index (kg/m^2^)	
< 25	92 (32%)
25–30	88 (30%)
30–35	56 (19%)
35+	56 (19%)
Blood Pressure - Mean (SD)	
Systolic	119 (14)
Diastolic	74 (10)
Daily Medications	
0	127 (43%)
1	89 (30%)
2+	76 (26%)
Number of Comorbidities	
0	116 (40%)
1+	151 (52%)
Did not provide	25 (9%)
Musculoskeletal Index - Mean (SD)	1.2 (1.8)
Walking Distance Limitations	
Unlimited walking (>20 blocks)	189 (66%)
Limited walking (<20 blocks)	98 (34%)
Team Formation Method	
Participant formed	233 (78%)
Study-formed	67 (22%)

### Fitbit wear adherence & physical activity

Ninety-four percent of participants wore the Fitbit ≥4 days at the end of the first month. By the end of the 6 months, 62% of the cohort wore the Fitbit ≥4 days. One hundred eighty-four study participants (63%) were adherent Fitbit wearers, wearing their Fitbit ≥4 days per week for at least 20 out of 24 intervention weeks.

**Fig. 1:**
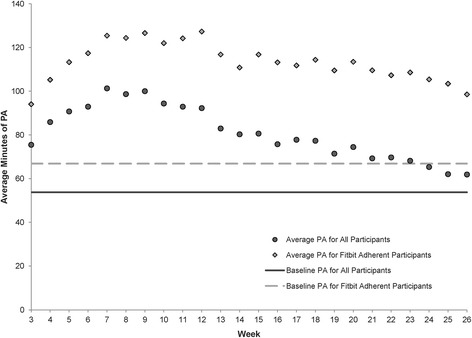
Average minutes of physical activity by week. The average minutes of physical activity at baseline and each week throughout the study are depicted for all study participants and for Fitbit adherent study participants. Fitbit adherence was defined as wearing the activity monitor ≥4 days per week for ≥20 weeks throughout the intervention. Baseline measures of physical activity were obtained during the two-week pre-intervention phase. The black circles show the average PA for all participants, and the light gray diamonds show the average PA for Fitbit adherent participants. The solid dark line shows the pre-intervention PA level for all participants, and the light dashed line shows the pre-intervention PA level for Fitbit adherent participants. Abbreviations: PA, physical activity

The 292 participants self-reported a mean of approximately 15 min of PA per week prior to the start of B-Well; half reported no exercise and half reported 30 min of exercise. During the initial two-week pre-intervention period, the average weekly duration of MVPA was 54 (SD 64) minutes. One month into the intervention, the average duration of MVPA increased to 93 (SD 87) minutes, and 72% of participants were above their personal pre-intervention baseline levels of MVPA. By week 14 (halfway through the intervention), average weekly MVPA was 80 (SD 90) minutes. At the end of the intervention, the average duration of weekly MVPA was 62 (SD 89) minutes, and 41% of participants were above their personal baseline levels of MVPA by an average of 16 min. Over the intervention period, the average weekly duration of MVPA was 85 (SD 76) minutes (Fig. 1).

Compared to Fitbit non-adherers, adherent Fitbit wearers had higher pre-intervention levels of MVPA and achieved higher intervention levels of MVPA. The pre-intervention average duration of MVPA for adherent participants was 67 (SD 71) minutes; at week 14 (halfway), the average duration of MVPA increased to 111 (SD 94) minutes. Adherent participants completed, on average, 99 (SD 96) minutes of weekly MVPA at the end of the study, with an average duration of weekly MVPA of 113 (SD 80) minutes throughout the intervention.

### Meeting goals & CDC guidelines

**Fig. 2:**
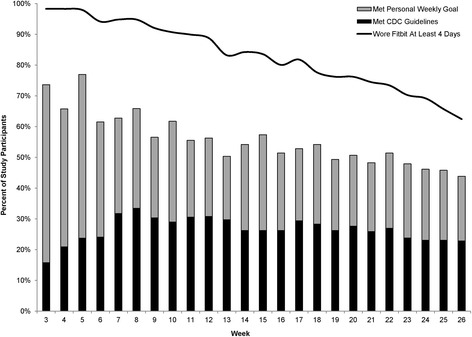
Percent of participants meeting personal goal or CDC guidelines, and percent wearing Fitbit at least 4 days of the week. This figure shows the proportion of the 292 participants meeting their personal goals or the CDC physical activity guidelines for each week of the study. The solid black line indicates the percent of participants wearing the Fitbit at least 4 days of the given week. Personal weekly goals were defined as a 10% increase in minutes of physical activity compared to the previous week. The total height of the bars represents the total percentage of participants who met either their personal weekly PA goals or the CDC physical activity guidelines. The black portions of the bars show the percent of participants meeting CDC guidelines, and the light gray portions show the percent of participants who met weekly PA goals, but not CDC guidelines. Participants without data were assumed to not have met guidelines or weekly goals

During the first week of the intervention, 58% of study participants increased their MVPA by at least 10% and met their weekly goal but did not meet CDC guidelines. An additional 16% of all participants met CDC guidelines. One month into the intervention, 37% of participants met their weekly goal but not CDC guidelines, and an additional 24% met CDC guidelines. By the end of the intervention, 23% of all participants met CDC guidelines (Fig. 2).

Eighty-six percent of the cohort met either their personal weekly goal or CDC guidelines for at least 6 individual weeks throughout the intervention period, and 52% met their goal or CDC guidelines for at least half of all intervention weeks. Six participants (2%) met their goal or CDC guidelines every week of the intervention.

**Fig. 3:**
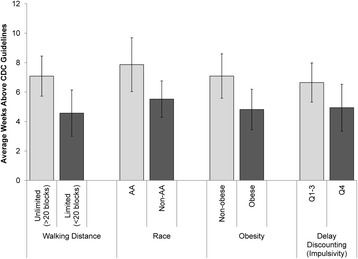
Average number of weeks meeting or exceeding CDC guidelines by baseline characteristic. The average (and 95% confidence interval) number of weeks that participants met or exceeded CDC physical activity guidelines is depicted for several baseline characteristics. Results are adjusted for age and comorbidities. Q4 is the highest quartile for delay discounting (immediate rewards preferred). Abbreviations: AA, African American

Across the 24 intervention weeks, participants met CDC guidelines an average of 6.3 weeks (SD 7.8). Multivariable analyses showed that reporting an ability to walk at least 20 blocks (one mile) at baseline, African American race, non-obesity, and lower delay discounting index scores were associated with an increased number of weeks meeting CDC PA guidelines (Fig. 3). Participants reporting the ability to walk >20 blocks at baseline achieved CDC guidelines an average of 7 weeks, while participants unable to walk 20 blocks at baseline achieved CDC guidelines an average of 5 weeks (*p* = 0.010). African Americans achieved CDC guidelines an average of 8 weeks, while all other races met CDC guidelines an average of 6 weeks (*p* = 0.016). Non-obese persons achieved CDC guidelines an average of 7 weeks throughout the duration of the study, while obese participants achieved CDC guidelines an average of 5 weeks (*p* = 0.018). Participants in the lowest three quartiles of the delay discounting index achieved CDC guidelines approximately 7 weeks, while those in the highest quartile (those who attach most value to immediate rewards) achieved CDC guidelines an average of 5 weeks (*p* = 0.068).

We did not find an association between the method of team formation (independent versus matched by study staff) and number of weeks meeting CDC guidelines. However, the within-team correlation was estimated to be 0.5, suggesting that having one team member reach CDC guidelines was associated with increased likelihood of other team members meeting CDC guidelines. Within-team correlation for minutes of MVPA increased over time, ranging from 0.06 for week 1 (pre-intervention) to 0.32 at week 24.

### Financial rewards and satisfaction

On average, participants earned $220 (SD $168) by meeting goals throughout the intervention. Three participants earned the maximum $860, and one participant did not achieve any goals and therefore did not receive any goal-based compensation.

Two hundred fifty-five out of 298 participants (86%) responded to the satisfaction survey. Sixty-six percent of participants were satisfied with the B-Well program overall, with 79% indicating that they would participate again if offered. The majority (72%) of participants thought that the program helped increase PA; 58% thought that being part of a team helped them increase their PA, and 73% of participants indicated that receiving financial incentives was beneficial for promoting activity. Those who were not satisfied were less likely to achieve daily PA goals and receive incentives.

## Discussion

We provided financial incentives for 6 months to promote PA among 292 sedentary hospital employees. Average weekly MVPA among study participants increased by approximately 70 min compared to self-reported, pre-study MPVA. Greater walking ability, African-American race, lack of obesity, and lower impulsivity were associated with more consistently achieving CDC-recommended PA levels.

The increase in MVPA in our study aligns with previous work on improving activity using financial rewards [22, 23, 33]. In a financial incentive program trial comparing individual, team-based, or combined (team and individual) structures, the combined financial incentive structure was the most successful at improving daily step counts. The individuals in the combined financial incentives arm reached their daily step goals nearly twice as often as those in the control group [23]. We found that combined individual and team-based financial incentives increased PA from pre-intervention levels. B-Well expands upon findings about daily steps by showing that financial incentives can successfully increase MVPA levels and promote adherence to CDC guidelines.

However, the gains in our program are smaller than gains reported by other financial incentive studies. B-Well participants on average met guidelines for 25% of program weeks. In a trial investigating methods of framing financial incentives, Patel and colleagues found that during the intervention, the percentage of participants achieving the goal of 7000 steps/day ranged from 30% (control) to 45% (loss-incentives) [22]. In a trial of team versus individual financial incentives, the percentage of days achieving the goal ranged from 18% (control) to 35% (combined team and individual incentives) [23]. These trials may have had higher success rates than B-Well because their goals were defined in steps/day, while B-Well goals were intensity based, making them more difficult to achieve. Moreover, the participants in these studies received daily feedback about their progress, which would allow them to track their progress more easily than the B-Well participants.

The greatest increase in MVPA was within B-Well’s first 6 weeks, when average MVPA levels increased by approximately 84 min compared to self-reported, pre-study MVPA. By the end of 6 months, MVPA levels were about 47 min higher, with the 184 (63%) Fitbit adherent participants sustaining higher MVPA throughout the study. This suggests that participants who engaged with the study were more likely to increase their MVPA. While it is possible that non-adherent subjects were achieving high levels of MVPA but not recording it, this seems unlikely, as this would be tantamount to declining the financial incentives.

A novel finding of B-Well was that participant impulsivity, measured by delay discounting index, was associated with physical activity. More impulsive participants met CDC guidelines for 2 weeks less than the less impulsive participants. This suggests that financial incentives, which provide an immediate reward to activity, do not abrogate the effect of impulsivity. Future studies should to compare with a control to determine if more impulsive subjects are a subgroup that may be more responsive to incentives.

Future studies should also investigate the extent of attrition after incentives have ended. Our limited follow-up precluded this analysis. The key theory underlying financial incentives suggests that incentives reinforce behavior formation; once a behavior becomes a habit, the incentives are no longer needed. But, natural attrition may follow incentive removal as incentives “crowd-out” intrinsic motivation [34]. There are no clear guidelines regarding the optimal duration of the ‘behavior formation’ stage or the amount of incentives that would facilitate behavior formation. The literature on behavior maintenance after incentives are removed is also inconclusive. Systematic reviews published in 2014 and 2015 found that long-term maintenance of increased activity had not been adequately studied [20, 35]. A subsequently published study found that while cash incentives were in effect, the incentives group outperformed a control in terms of minutes of MVPA. However, after the incentives ended, MVPA in the incentives arm reverted to baseline levels [33]. Other studies of incentives also observed a decline in activity once the incentives were stopped [22, 23]. Such data suggest that boosted interventions to optimize intrinsic and extrinsic motivation may be needed to improve the sustainability of interventions.

We note several limitations to B-Well. Participants were recruited from a single center and were predominantly college-educated, white females, which may limit generalizability. As the program did not include a control arm due to budgetary restrictions, we cannot conclude which elements – financial incentives, team structure, or PA monitoring – increased MVPA. Similarly, as we incorporated both individual and team-based rewards, we are unable to ascertain their respective independent effects. However, the high within-team correlation for MVPA suggests a strong team effect. During the pre-intervention weeks, average weekly MVPA was 54 min, substantially higher than the average of 15 min self-reported by participants before enrollment. This increased MVPA could be due to the activation effect of enrolling in the study and wearing the Fitbit. Thus, the pre-intervention period cannot be used as a fair assessment of baseline MVPA levels. It is also possible that participants underreported their MVPA levels to become eligible for the study. However, we did not disclose PA eligibility requirements prior to screening, making this less likely.

In addition, MVPA was only recorded while participants were wearing their Fitbits; therefore, adherence to Fitbit wear contributed to our results. The proportion of participants wearing their Fitbit for at least 4 days in the week ranged from nearly 100% in the first 2 weeks of the study to 62% by week 26. As there is more incentive to wear the Fitbit when financial incentives are in effect, our observed increase in MVPA could be attributable to increased wear-time. To avoid this, we paid participants for wearing the Fitbit during the pre-intervention period. Minutes of MVPA are not reported directly by Fitbit, so participants were unable to view their MVPA in real-time. This may have contributed to participant dissatisfaction, as participants did not know if they were on track to meet their goals until the deadline had passed. Lastly, the Fitbit Flex captures only ambulatory activity, so we were unable to account for other aerobic activity.

## Conclusions

B-Well results suggest that the program led to increased MVPA among inactive employees. Participants with adherent Fitbit wear had substantially higher MVPA over the course of the study than non-adherers. PA-enhancing programs should identify effective methods of incentivizing activity monitor adherence to enhance the PA gains of the intervention. Future studies should also assess methods for engaging non-adherent wearers in interventions. B-Well design and data can be used by employers and policymakers to guide the design of workplace programs aimed at increasing activity levels among employees.

## Abbreviations

B-Well: Brigham and women’s wellness initiative
CDC: Centers for Disease Control and Prevention
MVPA: Moderate-to-vigorous physical activity
PA: Physical activity
SD: Standard deviation
